# Supplementary material for: The genome sequence of a beetle, Brachypterus glaber (Newman, 1834)
Source: Wellcome Open Res. 2025 Jan 14;10:10. [Version 1] doi: 10.12688/wellcomeopenres.23459.1 (PMC11814963; doi:10.12688/wellcomeopenres.23459.1)
Supplement: Peer review history

# Reviewer report: Reviewer response for version 1

**DOI:** 10.21956/wellcomeopenres.25869.r117527

**Published:** 2025-02-11

**Authors:** Chenyang Cai

**Affiliations:** 1 Chinese Academy of Sciences, Nanjing, China

The manuscript presents a high-quality genome assembly of *Brachypterus glaber*, a member of Kateretidae, contributing to the growing genomic resources for Coleoptera. The study used advanced sequencing and assembly techniques to generate a comprehensive genomic dataset. The genome assembly exhibits a high degree of completeness. 

*B. glaber *at the beginning of a paragraph should be ' *Brachypterus glaber*'.

Are sufficient details of methods and materials provided to allow replication by others?

Yes

Is the rationale for creating the dataset(s) clearly described?

Yes

Are the datasets clearly presented in a useable and accessible format?

Yes

Are the protocols appropriate and is the work technically sound?

Yes

Reviewer Expertise:

insect diversity, phylogenomics, fossils

I confirm that I have read this submission and believe that I have an appropriate level of expertise to confirm that it is of an acceptable scientific standard.

# Reviewer report: Reviewer response for version 1

**DOI:** 10.21956/wellcomeopenres.25869.r117212

**Published:** 2025-02-04

**Authors:** James RM Bickerstaff

**Affiliations:** 1 CSIRO Consortium, Canberra, Australian Capital Territory, Australia

Overall, the paper is great with sufficient detail to understand what went into the genome assembly and how to replicate it. I have two questions regarding the methodology which could improve the accuracy of the methodologies, for complete repeatability.

 To run mitoHiFi, which reference mtGenomes were used?

 What protein database was used to annotate the genome in BRAKER2?

Are sufficient details of methods and materials provided to allow replication by others?

Partly

Is the rationale for creating the dataset(s) clearly described?

Yes

Are the datasets clearly presented in a useable and accessible format?

Yes

Are the protocols appropriate and is the work technically sound?

Yes

Reviewer Expertise:

genomics, comparative genomics, phylogenomics, taxonomy, systematics.

I confirm that I have read this submission and believe that I have an appropriate level of expertise to confirm that it is of an acceptable scientific standard.
